# Imipenem Resistance in *Clostridium difficile* Ribotype 017, Portugal

**DOI:** 10.3201/eid2404.170095

**Published:** 2018-04

**Authors:** Joana Isidro, Andrea Santos, Alexandra Nunes, Vítor Borges, Catarina Silva, Luís Vieira, Aristides L. Mendes, Mónica Serrano, Adriano O. Henriques, João Paulo Gomes, Mónica Oleastro

**Affiliations:** National Institute of Health, Lisbon, Portugal (J. Isidro, A. Santos, A. Nunes, V. Borges, C. Silva, L. Vieira, J.P. Gomes, M. Oleastro);; Instituto de Tecnologia Química e Biológica António Xavier, Oeiras, Portugal (A.L. Mendes, M. Serrano, A.O. Henriques)

**Keywords:** *Clostridium difficile* infection, CDI, imipenem resistance, *Clostridium difficile*, penicillin-binding proteins, ribotype 017, multidrug resistance, bacteria, antimicrobial resistance, Portugal

## Abstract

We describe imipenem-resistant and imipenem-susceptible clinical isolates of *Clostridium difficile* ribotype 017 in Portugal. All ribotype 017 isolates carried an extra penicillin-binding protein gene, *pbp5*, and the imipenem-resistant isolates had additional substitutions near the transpeptidase active sites of *pbp1* and *pbp3*. These clones could disseminate and contribute to imipenem resistance.

*Clostridium difficile*, a toxin-producing, spore-forming bacillus, is a main cause of nosocomial antimicrobial drug–associated diarrhea in industrialized countries (*1*). *C. difficile* infection (CDI) usually develops in previously hospitalized persons with a recent history of antimicrobial drug use and causes illness with symptoms ranging from mild diarrhea to potentially lethal pseudomembranous colitis (*2*). Antimicrobial drugs disrupt the protective gut microbiota, enabling ingested *C. difficile* spores to germinate in the colon and providing a selective advantage to nonsusceptible strains (*3*). CDI is mainly mediated by the TcdA and TcdB toxins, though some strains additionally produce a binary toxin. Multiple antimicrobial drugs can promote CDI, and cephalosporins and fluoroquinolones have been associated with a higher risk for CDI (*3*). Multidrug resistance is frequently found in epidemic *C. difficile* strains; determinants of resistance are often found in horizontally transferable mobile genetic elements (*4*). In past decades, CDI prominence has increased because of a sudden rise in outbreaks and an increase in disease severity and death (*5*). This shift was mainly associated with the dissemination of fluoroquinolone-resistant PCR ribotype (RT) 027, which has been responsible for hospital outbreaks worldwide. Strains of other ribotypes, including RT078 and RT017, which have enhanced virulence, have emerged (*6*). In particular, RT017, the most common toxin A–negative, toxin B–positive ribotype, is widespread in Asia and is common in Europe (*7*–*9*). In a pan-European study of ≈900 *C. difficile* strains, the overall rate of resistance to imipenem, an antimicrobial drug of the carbapenem class, currently widely used as a last-line drug to treat infections by gram-negative bacteria, was found to be 7.41%, and the geometric mean (GM) MIC of imipenem for RT017 strains was 5.91 mg/L (*8*). In another study, isolates collected in a South Korea hospital during 2000–2009 were analyzed, and a resistance rate to imipenem of 8% (12% among RT017 isolates) was found (*10*).

## The Study

We characterized 191 *C. difficile* isolates collected during September 2012–September 2015 from 15 hospitals in Portugal (Technical Appendix). We found 24 (12.6%) were resistant to imipenem. Of these 24 isolates, 22 were RT017, 1 was RT014, and 1 was RT477. The MIC for imipenem for RT017, the imipenem-resistant isolates, was >32 mg/L (Table 1); the MIC for the 2 non-RT017 isolates was 16 mg/L. The 22 imipenem-resistant RT017 isolates were found at hospital A throughout the study period, suggesting the existence of a persistent clone, a finding supported by whole-genome sequencing data (Technical Appendix). Among the 25 RT017 isolates, 3 were imipenem-susceptible and from hospital B (MIC range 1.5–3 mg/L) (Table 1).

**Table 1:** Susceptibility of *Clostridium difficile* RT017 imipenem-resistant isolates from hospital A and imipenem-susceptible isolates from hospital B to 11 antimicrobial drugs, Portugal*

Hospital	Resistance breakpoint†	Antimicrobial drug, MIC breakpoints, mg/L										
		IMP‡	ETP‡	MRP‡	MXF†§	MTZ†§	VAN†§	CLI‡	CHL‡	RIF†	TGC†	TET‡
>16	>16	>16	>4	>2	>2	>8	>32	>0.004	>0.25	>16
A, 22 isolates	MIC range	>32	3–16	1.5–4	>32	<0.016–1	0.38–2	>256	2–6	>32	<0.016–0.094	16–32
	GM MIC	32	7.56	2.31	32	0.12	0.73	256	3.29	32	0.025	18.08
	MIC_90_	32	12	3	>32	0.38	2	256	4	32	0.032	32
	MIC_50_	32	6	2	>32	0.19	0.75	256	3	32	0.023	16
	% Resistant	100	4.5	0	100	0	0	100	0	100	0	100
B, 3 isolates	MIC range	1.5–3	1.5–2	0.5–1.5	1.5	<0.016–0.25	0.38–0.75	>256	3–4	>32	<0.016–0.023	16
	GM MIC	2.08	1.82	0.83	1.5	0.072	0.60	256	3.30	32	0.020	16
	MIC_90_	3	2	1.5	1.5	0.25	0.75	256	4	32	0.023	16
	MIC_50_	2	2	0.75	1.5	0.094	0.75	256	3	32	0.023	16
	% Resistant	0	0	0	0	0	0	100	0	100	0	100
p value		<0.0001	<0.0001	<0.0001	<0.0001	0.45	0.56	ND	0.98	ND	0.41	0.51

*CHL, chloramphenicol; CLI, clindamycin; ETP, ertapenem; GM, geometric mean; IMP, imipenem; MIC_50_, minimal inhibitory concentration for 50% of strains; MIC_90_, minimal inhibitory concentration for 90% of strains; MRP, meropenem; MTZ, metronidazole; MXF, moxifloxacin; ND, not done; RIF, rifampin; TGC, tigecycline; VAN, vancomycin. †European Committee on Antimicrobial Susceptibility Testing breakpoint. ‡Clinical and Laboratory Standards Institute breakpoint. §Previously determined (*9*).

**Figure 1:**
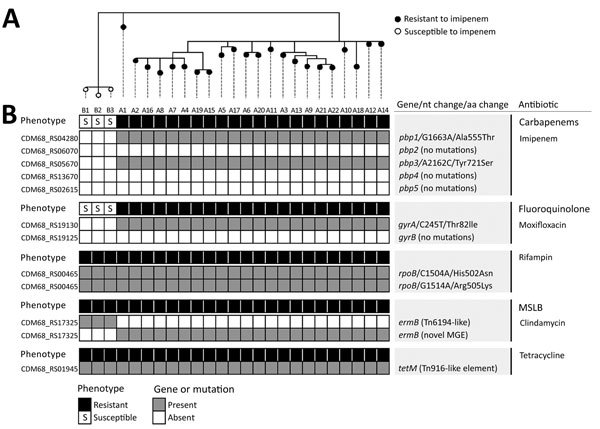
Phylogeny of *Clostridium difficile* RT017 isolates from hospitals A and B and genetic determinants of antimicrobial drug resistance, Portugal. A) Core genome single-nucleotide polymorphism–based neighbor-joining phylogeny of 25 RT017 *C. difficile* clinical isolates reconstructed by using 47 variant sites (outside MGEs) identified when mapping to either the corresponding genomic sequence of close relative *C. difficile* strain M68 (GenBank accession no. NC_017175) or a draft genome sequence of a representative clinical isolate. B) For each isolate, the profile of antimicrobial drug susceptibility is indicated together with respective potential genetic determinants of antimicrobial drug resistance. Only antimicrobial drugs for which a resistant phenotype was observed are displayed. Gene locus tags are relative to the *C. difficile* M68 genome annotation. Both nucleotide and amino acid replacements refer to mutations in the resistant isolates when comparing with susceptible isolates. No mutations means that no mutations are present differentiating resistant isolates of hospital A from susceptible isolates of hospital B, although mutations are present relative to M68. Both the *pbp5*-carrying region and the *ermB* gene (present in all isolates) were found to be inserted in distinct genomic contexts (Technical Appendix). MSLB, macrolide/lincosamide/streptogramin B; MGE, mobile genetic element.

RT017 *C. difficile* strains are frequently resistant to clindamycin, erythromycin, moxifloxacin, tetracycline, or rifampin (individually or in combination) (*8*,*10*). In this study, the 22 RT017 imipenem-resistant isolates were also found to be resistant to all of these antimicrobial drugs and showed higher meropenem and ertapenem MICs than those of the RT017 imipenem-susceptible isolates (Table 1; Technical Appendix; Technical Appendix Figure). Multidrug resistance to noncarbapenem antimicrobial drugs correlated with the presence of several genetic determinants, many located in mobile genetic elements (Figure 1; online Technical Appendix), in line with the idea that multidrug-resistant strains have a selective advantage (*4*) and that horizontal gene transfer plays a major role in the evolution of this pathogen (*11*).

**Figure 2:**
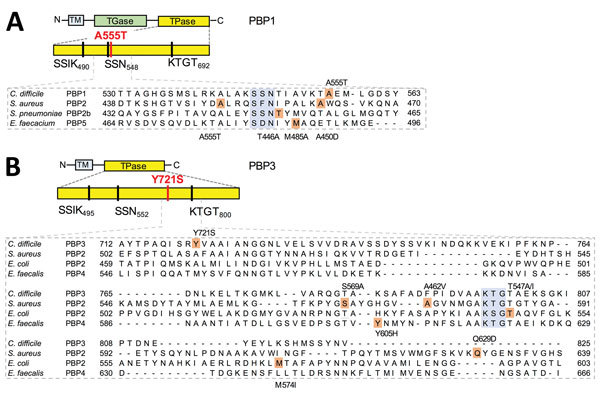
Amino acid substitutions in 2 PBPs predicted to be associated with imipenem resistance in *Clostridium difficile*, Portugal. The domains and conserved motifs SXXK, SXN, and KTG[T/S] are shown for the following proteins: PBP1 (A), homolog of CDM68_RS04280 of RT017 strain M68 (GenBank accession no. NC_017175) or CD630_07810 in the laboratory strain 630; and PBP3 (B), homolog of CDM68_RS05670 or CD630_11480. The mutations found in these resistant isolates are marked by red lines. The alignments below the 2 proteins show the position (shaded in pink) and nature of the amino acid substitutions observed in the imipenem-resistant RT017 isolates and select PBPs from microorganisms *Staphylococcus aureus* (GenBank accession no. AAA74375.1), *Streptococcus pneumoniae* (GenBank accession no. WP_001829432.1), *Escherichia coli* (GenBank accession no. AAB40835.1), *Enterococcus faecalis* (GenBank accession no. AAS77615.1), and *Enterococcus faecacium* (GenBank accession no. AIG13039.1). The conserved motifs in the vicinity of the substitutions are shaded in blue. PBP, penicillin-binding protein; TGase, transglycosylase; TM, transmembrane; TPase, transpeptidase.

Through whole-genome sequencing, we found 13 single-nucleotide polymorphisms (SNPs) that differentiated the imipenem-resistant and imipenem-susceptible RT017 isolates (Table 2; Figure 1; Technical Appendix). We found 2 SNPs in the genes coding for 2 high molecular weight (HMW) penicillin-binding proteins (PBPs) (Figure 1). HMW PBPs, which are bifunctional enzymes containing transglycosylase and transpeptidase domains, are categorized into class A, and PBPs lacking the transglycosylase domain are categorized into class B. The transpeptidase domain harbors 3 functional motifs (SXXK, SXN, and KTG[T/S]) that comprise the active site. Carbapenems block cell wall synthesis by inhibiting transpeptidase activity (*12*). One of the mutations found in the imipenem-resistant isolates affected the gene coding for PBP1, the single class A bifunctional peptidoglycan synthase of *C. difficile*; the mutation resulted in the amino acid substitution Ala555Thr close to the SSN functional motif (Figure 2). The second mutation was found in the gene encoding for the PBP3 class B transpeptidase and caused the amino acid replacement Tyr721Ser between the SXN and KTGT motifs (Figure 2). Neither of these changes was found in the 3 imipenem-susceptible RT017 isolates (Figure 1). Moreover, the 2 non-RT017 imipenem-resistant isolates, with a MIC for imipenem lower than that of the RT017 isolates, revealed either the Ala555Thr change or a different substitution (Leu543His), both in PBP1, also close to the functional motif SXN (Table 2). Modified PBPs with reduced affinity for the antimicrobial drug have been associated with resistance to β-lactams and specifically to imipenem in several microorganisms (*12*). We found no differences between the imipenem-resistant and imipenem-susceptible RT017 isolates in genes encoding other peptidoglycan synthases (Figure 1). It is possible that the substitutions in PBP1 and PBP3 in RT017 confer high-level resistance to imipenem and reduced susceptibility to other carbapenems, and at least in the RT014 and RT477 isolates studied, the single Ala555Thr substitution (or other substitutions in the vicinity of the SXN motif) is sufficient for an intermediate level of resistance.

**Table 2:** Mutations differentiating *Clostridium difficile* RT017 imipenem-resistant isolates found at hospital A from imipenem-susceptible isolates found at hospital B, Portugal

Gene in M68 genome*	Genome position*	Nucleotide in M68	Nucleotide change†	Amino acid change†	Gene product
RS02665	512416	C	C578T	Ala193Val‡	Multidrug ATP-binding cassette transporter permease, associated with antimicrobial drug resistance
RS04280/*pbp1*	905394	G	G1663A	Ala555Thr‡	Penicillin-binding transpeptidase
RS04935	1048151	C	T1010C	Ile337Thr‡	3-Isopropylmalate dehydratase large subunit
RS05670/*pbp3*	1221182	G	A2162C	Tyr721Ser‡	Penicillin-binding protein
RS07765	1666351	G	G214T	Gly72§	Hypothetical protein
RS07795/*hisB*	1671129	T	T209C	Ile70Thr‡	Imidazoleglycerol-phosphate dehydratase
RS07810	1673280	T	C474T	Ala158Ala	Imidazoleglycerol-phosphate synthase cyclase subunit
RS08415	1792079	G	A241G	Lys81Glu‡	Hypothetical protein (domain of MerR-like transcriptional regulators)
RS08810	1882950	C	C420T	Asp140Asp	Flavodoxin
RS14235	3083548	G	G421T	Gly141§	Haloacid dehalogenase
RS18530	4054525	C	C220T	Gln74§	S-adenosyl methionine–dependent methyltransferase
RS19130/*gyrA*	4174650	C	C245T	Thr82Ile‡	DNA gyrase subunit A
RS19545	4255124	C	C400T	His134Tyr‡	Phage portal, SPP1 Gp6-like family protein

*Relative to the annotation of the *C. difficile* M68 genome (GenBank accession no. NC_017175). †Changes observed between imipenem-resistant and imipenem-susceptible isolates.  ‡Nonsynonymous mutations. §Mutations leading to putative protein truncation.

However, all RT017 isolates studied herein, as well as the previously annotated strains M68 (GenBank accession no. NC_017175) and BJ08 (accession no. CP003939), have a fifth HMW class B PBP, PBP5, encoded in a mobile element (Technical Appendix). Whether PBP5 contributes to imipenem resistance remains to be determined. Moreover, in imipenem-resistant isolates, the key sporulation-specific gene *sigK*, which is contiguous to *pbp2*, is interrupted by the 17-kb *skin*^cd^ element (*13*), and the *pbp5* region is contiguous to a transposon-like element carrying the *ermB* gene (shown as PUBMLST allele 8; https://pubmlst.org/bigsdb?db=pubmlst_cdifficile_seqdef&page=alleleInfo&locus=ermB&allele_id=8). It is unknown whether these genetic differences contribute to imipenem resistance.

## Conclusions

Imipenem resistance in *C. difficile* RT017 probably involves the acquisition of mutations in both *pbp1* and *pbp3* that lead to amino acid substitutions close to the functional motifs of their transpeptidase domains. These substitutions might decrease the affinity of PBP1 and PBP3 for imipenem, enabling peptidoglycan synthesis in the presence of the antimicrobial drug. Considering that the presence of an additional PBP (PBP5) is a characteristic of RT017 strains, we suggest that PBP5 facilitates the expression of imipenem resistance through acquisition of mutations in *pbp1* and *pbp3*. In strains of other ribotypes lacking PBP5, such as the RT014 and RT477 isolates herein described, mutations in *pbp1* might only lead to intermediate levels of resistance. We further suggest that the spreading of *pbp5* might contribute to the dissemination of high-level imipenem-resistance.

Portugal has a high rate of healthcare-associated infections and is a major consumer of carbapenems (*1*). Although carbapenem consumption has not been directly linked to *C. difficile* resistance, we speculate that the emergence of resistance and reduced susceptibility to these antimicrobial drugs might recapitulate the scenario observed with fluoroquinolone-resistant RT027 in the United States, where fluoroquinolones were the most prescribed antimicrobial drug (*14*). Our findings further reinforce the need for the responsible use of antimicrobial drugs; the emergence of carbapenem resistance in multidrug-resistant *C. difficile* clones might result in the dissemination of resistant strains.

Technical Appendix Materials and methods, additional results and discussion of antimicrobial resistance profiles and genetic determinants of imipenem resistance in *Clostridium difficile* isolates, primer sequences for sequencing transpeptidase region of penicillin-binding protein genes, and carbapenem susceptibility profile of *C. difficile* ribotype 017 isolates.
