# Supplementary material for: Imipenem Resistance in Clostridium difficile Ribotype 017, Portugal
Source: Emerg Infect Dis. 2018 Apr;24(4):741–5. doi: 10.3201/eid2404.170095 (PMC5875251; doi:10.3201/eid2404.170095)
Supplement: Technical Appendix — Materials and methods, additional results and discussion of antimicrobial resistance profiles and genetic determinants of imipenem resistance in Clostridium difficile isolates, primer sequences for sequencing transpeptidase region of penicillin-binding protein genes, and carbapenem susceptibility profile of C. difficile ribotype 017 isolates. [file 17-0095-Techapp-s1.pdf]

Article DOI: https://doi.org/10.3201/eid2404.170095

Imipenem Resistance in *Clostridium difficile* Ribotype 017, Portugal

Technical Appendix

Materials and Methods

Clostridium difficile Strains

A group of 191 strains isolated during 2012–2015 from 15 Portuguese hospitals (*1*) were tested for imipenem susceptibility. The group included strains from the ribotypes most commonly found in Portugal, including ribotype (RT) 027 (n = 33), RT017 (n = 25), RT014 (n = 16), RT203 (n = 9), RT126 (n = 8), RT078 (n = 5), and RT020 (n = 3). The remaining 92 isolates comprised 51 less common ribotypes (*1*).

Antimicrobial Susceptibility Testing

MIC of imipenem was determined with Etest strips (bioMérieux, Marcy l'Etoile, France) on brucella blood agar with hemin and vitamin K1 (Becton Dickinson, Heidelberg, Germany), according to the manufacturer´s instructions. Considering that microcolonies were found inside the inhibition ellipse (MIC >32 mg/L) at 48 h of growth but not at 24 h for most susceptible strains, MICs of imipenem were read at 24 h to avoid false resistance. Subculturing of these microcolonies yielded the same susceptible profile, which was further confirmed by the agar dilution method. For control purposes, the agar dilution method was also performed for 10 resistant strains (8 RT017, 1 RT014, and 1 RT477 isolates) and 45 susceptible strains (multiple RTs) to confirm resistant and susceptible phenotypes.

The imipenem resistant isolates identified were subsequently tested against other carbapenems (meropenem and ertapenem) and also against rifampin, clindamycin, chloramphenicol, tetracycline, and tigecycline. Resistance to imipenem was confirmed by the agar dilution method on Wilkins-Chalgren (Oxoid, Basingstoke, UK) agar, as described by Freeman et al., with imipenem at 8 mg/L, 16 mg/L, and 32 mg/L, plus drug-free control plates (*2*). The EUCAST (European Committee on Antimicrobial Susceptibility Testing) and CLSI

(Clinical and Laboratory Standards Institute) breakpoints used are presented in Table 1 of main text.

Whole-Genome Sequencing and Data Analysis

Genomic DNA was extracted from pure cultures of 25 *Clostridium difficile* strains by using the Isolate II Genomic DNA kit (Bioline, London, UK). For each strain, whole-genome sequencing (WGS) was carried out as previously described (*3*). For each strain, draft genome sequences were de novo assembled by using Velvet (version 1.2.10) (*4*) with runs optimized taking advantage of VelvetOptimiser script version 2.2.5 (http://www.vicbioinformatics.com/software.velvetoptimiser.shtml). Draft genome sequences were analyzed to do the following: a) perform in silico multilocus sequence typing (MLST) and allele determination of well-known virulence-associated genes by using the online platform available at PUBMLST (http://pubmlst.org/); b) search for the presence of putative antimicrobial resistance (AMR) genes by using both CARD (https://card.mcmaster.ca/) and ResFinder (http://www.genomicepidemiology.org/); c) identify potential dissimilarities enrolling AMR genes; and d) verify the genomic context of potential horizontally transferable AMR genes. To identify mutations likely associated with imipenem resistance, 2 core genome SNP-based approaches were followed: a de novo assembly strategy with Harvest (*5*) and an assembly-free strategy with Snippy v3.1 (https://github.com/tseemann/snippy). To potentiate the use of high-quality SNPs, only variant calls passing default criteria (*5*) were considered in the Harvest approach, while for Snippy, only variant sites with minimum mapping quality of 60, minimum number of reads covering the variant position of 10, and minimum proportion of reads differing from the reference of 90% were considered for downstream analysis. For the Snippy approach, reads were initially mapped against the most closely related *C. difficile* genome available at GenBank (strain M68; accession no. NC_017175). To maximize the number of core genome sites available for SNP comparison, core genome SNP-based analyses were repeated by using a draft assembled genome of 1 representative clinical strain (isolate B2) as a reference sequence. MEGA5 software (http://www.megasoftware.net) was applied to calculate matrices of nucleotide distances and perform phylogenetic reconstructions over the obtained core genome SNP alignment by using the neighbor-joining method with bootstrapping (1,000 replicates). Non-RT017 imipenem-resistant strains were subjected to PCR and Sanger sequencing (Technical Appendix Table) as a matter of scrutiny for *pbp*-associated mutations found for the RT017

isolates. Raw sequence reads of the 25 *C. difficile* isolates subjected to WGS were deposited in Sequence Read Archive under the accession nos. SRR4199259, SRR4199346, SRR4199859, SRR4201714, SRR4205841, SRR4205953, SRR4205954, SRR4213076, SRR4213123, SRR4237569, SRR4237571, SRR4237665, SRR4237666, SRR4237667, SRR4238391, SRR4238392, SRR4238569, SRR4240476, SRR4240477, SRR4240494, SRR4240495, SRR4240496, SRR4240497, SRR4240498, and SRR4240499.

Results and Discussion

Antimicrobial Drug Resistance Profiles

Among the 191 *C. difficile* studied, 24 (12.6%) were resistant to imipenem (at 24 h of growth), of which 22 were RT017 strains (MIC >32 mg/L), 1 was RT014, and 1 was RT477 (MIC of 16 mg/L) (Table 1 in main text). Intermediary resistance was observed for 27 strains (14.1%; MIC range 6–12 mg/L), most of which (24 isolates) were of RT027. RT017 strains here identified to be imipenem-resistant had been previously found to be resistant to moxifloxacin, with a MIC of >32 mg/L, while the 3 selected imipenem-susceptible strains were found to be susceptibility to moxifloxacin (Table 1 main text). Both groups had also been found to be susceptible to metronidazole and vancomycin (*1*).

In the present study, further susceptibility testing against other carbapenems (Technical Appendix Figure) showed that imipenem-resistant strains exhibited a geometric mean (GM) MIC of 7.56 mg/L for ertapenem, with 17 presenting an intermediate MIC of 4–16 mg/L and 1 being resistant (MIC of 16 mg/L) (Technical Appendix Figure). The imipenem-susceptible strains were susceptible to ertapenem (GM MIC of 1.82 mg/L). All strains were susceptible to meropenem, although the imipenem-resistant strains had a significantly higher GM MIC (2.31 mg/L) when compared with the imipenem-susceptible strains (GM MIC of 0.83 mg/L) (Figure 1 in main text; Table 1 in main text; Technical Appendix Figure). Both groups of strains were highly resistant to rifampin (MIC_{50} of 32 mg/L) and clindamycin (MIC_{50} of 256 mg/L), while resistance to tetracycline was borderline (MIC_{50} of 16 mg/L). All strains were susceptible to chloramphenicol and tigecycline.

Potential Genetic Determinants of Imipenem Resistance

WGS data was first subjected to in silico MLST analysis and evaluation of well-known *C. difficile* virulence-associated genes. All 25 RT017 strains belonged to sequence type 37 (MLST clade 4). The pathogenicity locus (PaLoc) showed a complete *tcdB* gene (PubMLST allele 9) but a disrupted *tcdA* gene, characteristic of this ribotype (*6*). No mutations were found in the putative toxin-negative regulatory gene *tcdC* (PubMLST allele 7, https://pubmlst.org/bigsdb?db=pubmlst_cdifficile_seqdef&page=alleleInfo&locus=tcdC_complete&allele_id=7) or in the *tcdE* gene, encoding holin-like protein. The transcriptional regulator *tcdR* is predicted to be functional, contrarily to the closest related strain (M68). Genes *cdtA* and *cdtB* were absent from all the genomes.

Core genome SNP-based analysis confirmed that the 3 RT017 susceptible strains from hospital B were separated in a single cluster apart from the 22 imipenem-resistant strains from hospital A (Figure 1 in main text). All strains revealed a large core genome sequence identity, with only 13 variant sites perfectly discriminating the imipenem-resistant from the imipenem-susceptible strains (Table 2 in main text). The imipenem-resistant strains had a G to A nucleotide substitution at position 1,663 (relative to the start codon) of *pbp1*, a homologue of the CDM68_RS04280, that results in the amino acid substitution Ala555Thr near the conserved SSN motif. They also showed an A to C mutation at position 2,162 in *pbp3*, a homologue of CDM68_RS05670, leading to the amino acid replacement Tyr721Ser located between the conserved SCN and KTGT transpeptidase motifs (Figure 2 in main text). These 2 *pbp* mutations are likely genetic determinants of imipinem resistance in *C. difficile* (see main text). Among the other 11 group-specific mutations, we highlight the well-known substitution Thr82Ile in *gyrA* (PubMLST allele 35, https://pubmlst.org/bigsdb?db=pubmlst_cdifficile_seqdef&page=alleleInfo&locus=gyrA&allele_id=35), which could be associated with the resistance to fluoroquinolones displayed by the 22 hospital A strains. The remaining 10 SNPs (8 nonsynonymous mutations) differentiating the 2 groups (Table 2 in main text) affected genes belonging to different functional categories, such as metabolic enzymes, a transcriptional regulator, a hypothetical protein, and a multidrug ATP-binding cassette transporter permease. Other penicillin-binding protein (PBP)–encoding genes revealed no differences among the 25 isolates of RT017 (Figure 1 in main text).

Additional Differences between Imipenem-Resistant and Susceptible Strains

The genomic architecture surrounding CDM68_RS06070 (here designated as *pbp2*) was found to differ between imipenem-resistant and imipenem-susceptible isolates. In the resistant isolates, *sigK* (contiguous to *pbp2)* is interrupted by the 17-kb *skin*^{cd} element, a previously described prophage-like insertion (*7*) highly similar in both genetic content and organization to that of the M68 strain. Of note, this element includes the *vanZ* gene, which is related to teicoplanin resistance. We note, however, that although important for the timely activity of the regulatory mother cell–specific sigma factor σ^K, *skin*^{cd} is dispensable for sporulation and has not been associated with β-lactam resistance (*8*).

On the other hand, CDM68_RS02615, herein referred to as *pbp5*, which has not been found in ribotypes other than RT017 (*C. difficile* usually has genes coding for only 4 high molecular weight PBP genes), is located inside a large region that displays traces of horizontal gene transfer (flanked by multiple repeat regions and containing genes coding for recombinases, integrases, and other phage-related proteins). Although all RT017 isolates carry the *pbp5* gene, in imipenem-resistant isolates (but not in the imipenem-susceptible isolates), the *pbp5* region is contiguous to a transposon-like element carrying the *ermB* gene (PUBMLST allele 8), which confers resistance to the MLS_B (macrolide, lincosamide, streptogramin B) class of antimicrobials. This *ermB*-containing element is likely rare, considering it has only been found in 1 of the available *C. difficile* genomes (strain F253; accession nos. NZ_AVKO01000052.1 and AVKO01000420.1). A single copy of *ermB* was also identified in the imipenem-susceptible isolates (PUBMLST allele 1) but located in a previously described Tn6194-like mobile element, similarly to the genome of strain M68 (genome position 3,779,408–3,806,743).

Genotype–phenotype associations were also found for other antimicrobial drugs tested (Figure 1 in main text). Indeed, all strains displayed 2 nucleotide mutations in *rpoB* (PubMLST allele 20, https://pubmlst.org/bigsdb?db=pubmlst_cdifficile_seqdef&page=alleleInfo&locus=rpoB&allele_id=20) conferring resistance to rifampin. The *tetM* gene (PUBMLST allele 15, https://pubmlst.org/bigsdb?db=pubmlst_cdifficile_seqdef&page=alleleInfo&locus=tetM&allele_id=15), which confers resistance to tetracycline, was found to be present in all 25 strains, integrated in a Tn916-like element. Additional in silico screening for antimicrobial resistance genes revealed the presence of a gene encoding a chloramphenicol acetyltransferase

(CDM68_02605 homologue) in all strains. However, its presence was not associated with resistance to chloramphenicol because all strains were susceptible to this antimicrobial drug.

References

1. Santos A, Isidro J, Silva C, Boaventura L, Diogo J, Faustino A, et al. Molecular and epidemiologic study of *Clostridium difficile* reveals unusual heterogeneity in clinical strains circulating in different regions in Portugal. Clin Microbiol Infect. 2016;22:695–700. PubMed http://dx.doi.org/10.1016/j.cmi.2016.04.002

2. Freeman J, Stott J, Baines SD, Fawley WN, Wilcox MH. Surveillance for resistance to metronidazole and vancomycin in genotypically distinct and UK epidemic *Clostridium difficile* isolates in a large teaching hospital. J Antimicrob Chemother. 2005;56:988–9. PubMed http://dx.doi.org/10.1093/jac/dki357

3. Borges V, Nunes A, Sampaio DA, Vieira L, Machado J, Simões MJ, et al. *Legionella pneumophila* strain associated with the first evidence of person-to-person transmission of Legionnaires' disease: a unique mosaic genetic backbone. Sci Rep. 2016;6:26261. PubMed http://dx.doi.org/10.1038/srep26261

4. Zerbino DR, Birney E. Velvet: algorithms for de novo short read assembly using de Bruijn graphs. Genome Res. 2008;18:821–9. PubMed http://dx.doi.org/10.1101/gr.074492.107

5. Treangen TJ, Ondov BD, Koren S, Phillippy AM. The Harvest suite for rapid core-genome alignment and visualization of thousands of intraspecific microbial genomes. Genome Biol. 2014;15:524. PubMed http://dx.doi.org/10.1186/s13059-014-0524-x

6. King AM, Mackin KE, Lyras D. Emergence of toxin A-negative, toxin B-positive *Clostridium difficile* strains: epidemiological and clinical considerations. Future Microbiol. 2015;10:1–4. PubMed http://dx.doi.org/10.2217/fmb.14.115

7. Haraldsen JD, Sonenshein AL. Efficient sporulation in *Clostridium difficile* requires disruption of the σ^K gene. Mol Microbiol. 2003;48:811–21. PubMed http://dx.doi.org/10.1046/j.1365-2958.2003.03471.x

8. Serrano M, Kint N, Pereira FC, Saujet L, Boudry P, Dupuy B, et al. A recombination directionality factor controls the cell type-specific activation of σ^K and the fidelity of spore development in *Clostridium difficile*. PLoS Genet. 2016;12:e1006312. PubMed http://dx.doi.org/10.1371/journal.pgen.1006312

Technical Appendix Table. Primers designed for amplification and sequencing of the transpeptidase region of the 2 *Clostridium difficile* penicillin-binding proteins genes mutated in the imipenem-resistant isolates, Portugal*

Gene†	Primer	Sequence	Product size, bp	Annealing temperature, °C
CDM68_RS04280	PBP1-F	5′-TTAGATGACCCTACTCAAGTGGAC-3′	1,482	53
and CD630_07810	PBP1-R	5′-AGAGCCAGAAGATTGCTTTCCT-3′		
	PBP1-SEQ†	5′-TGGTGGTCTAATTGTAAACACAAC-3′	NA	NA
CDM68_RS05670	PBP3-F	5′-CTGAGTGCCAAAGAAGCGTT-3′	2,134	52
and CD630_11480	PBP3-R	5′-CTGCTGGAGCAAAAGCAACA-3′		
	PBP3-SEQ†	5′-GCAGGAAAAGCACAGTCAGC-3′	NA	NA

*NA, not application; PBP, penicillin-binding protein.
†Primers used only for sequencing.

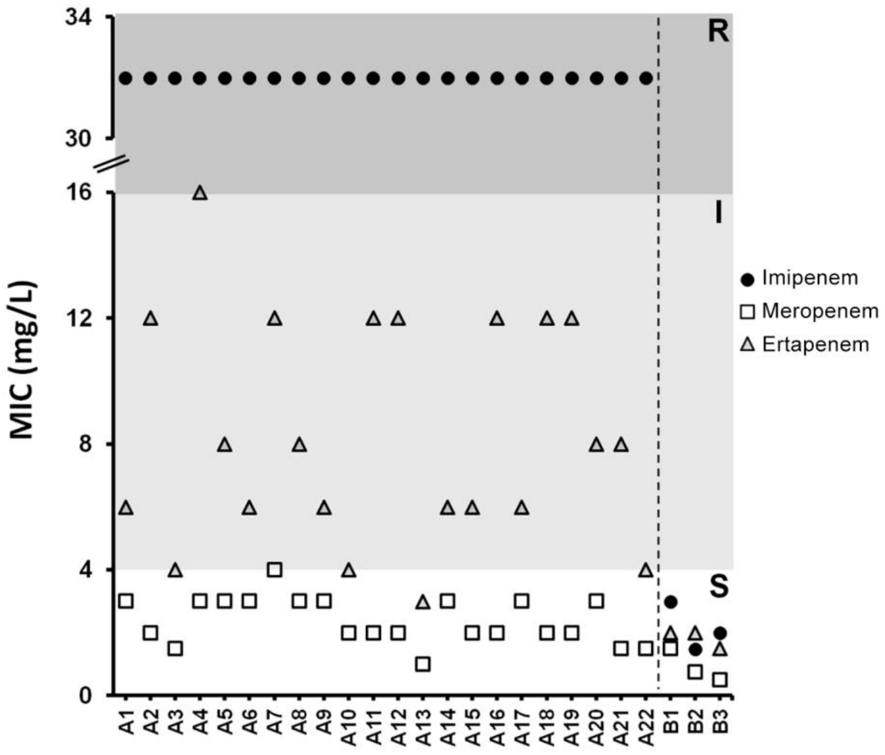

Technical Appendix Figure. Carbapenem susceptibility profile of the *Clostridium difficile* ribotype 017 isolates from hospitals A and B, Portugal. The graph shows the MIC values (mg/L) of each carbapenem tested (imipenem, meropenem, and ertapenem) for the 25 ribotype 017 isolates. MIC ranges, according to CLSI (Clinical and Laboratory Standards Institute) breakpoints, are also shown. Imipenem-resistant and imipenem-susceptible isolates are separated by a vertical dashed line. I, intermediate; R, resistant; S, susceptible.
